# Associations between pinch strength, cardiovascular events and all-cause mortality in patients undergoing maintenance hemodialysis

**DOI:** 10.1186/s12882-024-03587-x

**Published:** 2024-05-02

**Authors:** Yaqi Yang, Lin Liu, Yuzhuo Li, Rongshao Tan, Xiaoshi Zhong, Yun Liu, Yan Liu

**Affiliations:** 1 Clinical Collage of Medicine, Guizhou Medical University, Guiyang, China; 2 Department of Nephrology, Guangzhou Eighth People’s Hospital, Guangzhou Medical University, Guangzhou, China; 3 Department of Nephrology, Institute of Disease-Oriented Nutritional Research, Guangzhou Red Cross Hospital, Jinan University, Guangzhou, China

**Keywords:** Pinch strength, Handgrip strength, Cardiovascular events, All-cause mortality, Maintenance hemodialysis

## Abstract

**Background and aims:**

Patients undergoing maintenance hemodialysis (MHD) experience increased mortality and cardiovascular disease (CVD) risks; however, the potential connection between pinch strength (PS) and the prognosis of these patients remains unknown. Consequently, this study aimed to comprehensively assess the influence of PS and handgrip strength (HGS) on both survival and cardiovascular events (CVE) in patients undergoing MHD.

**Methods:**

Data were gathered from patients undergoing MHD at the Hemodialysis Center of Guangzhou Red Cross Hospital in March 2021. We performed a retr*o*spective follow-up spanning 24 months, with death serving as the primary endpoint for observation and CVE as the secondary endpoint. Multifactorial Cox regression analysis, Kaplan–Meier survival curves, trend tests, and restricted cubic spline were applied to explore the association.

**Results:**

During a 24-month follow-up, data were collected from 140 patients undergoing MHD with an average age of 66.71 ± 12.61 years. Among them, 52 (37.14%) experienced mortality, whereas 36 (40.00%) had CVE without baseline CVD. Kaplan–Meier survival curves demonstrated better survival rates and reduced CVE risk for patients in the second, third, and fourth quartiles compared with those in the first quartile for PS. Adjusted analyses in different models revealed higher PS levels were independently associated with all-cause mortality (major model, model 4, HR, 0.78; 95% CI, 0.64–0.95) but not with CVE risk (unadjusted HR, 0.90; 95% CI, 0.77–1.05). Compared with lower quartile PS levels, higher PS levels significantly reduced all-cause mortality (HR, 0.31; 95% CI, 0.10–1.02), and this trend remained consistent (P for trend = 0.021). Finally, the restricted cubic spline method using different models showed a linear relationship between PS and all-cause mortality (*P* > 0.05), when PS exceeded 4.99 kg, the all-cause mortality of MHD patients significantly decreased.

**Conclusions:**

PS was independently associated with all-cause mortality but not with CVE in patients undergoing MHD.

**Supplementary Information:**

The online version contains supplementary material available at 10.1186/s12882-024-03587-x.

## Introduction

The incidence of end-stage kidney disease (ESKD) is steadily rising worldwide. Hemodialysis is the primary treatment for patients with ESKD, accounting for approximately 69% of renal replacement therapy and 89% of dialysis cases [1]. Despite recent medical technological advancements enhancing the quality of life of patients undergoing maintenance hemodialysis (MHD), a notable mortality rate persists [2]. In particular, cardiovascular disease (CVD) has emerged as the leading cause of death in this population, with patients undergoing MHD facing a significantly elevated CVD mortality risk, approximately 10–30 times higher than that of the general population [3].

Sarcopenia is especially prevalent in patients undergoing MHD and is a significant predictor of all-cause mortality and cardiovascular events (CVE) [4]. Pinch strength (PS) and handgrip strength (HGS) are straightforward and objective metrics for assessing muscle strength [5], and are closely associated with overall health. HGS is a strong predictor of various health outcomes and reflects nutritional status [6] in populations both undergoing and not undergoing dialysis. Thomas J Wilkinson et al. presented proposals to use HGS as a surrogate indicator of protein energy status and functional status [7]. In the Prospective Urban Rural Epidemiology study, HGS exhibited a significant predictive value for all-cause mortality, cardiovascular mortality, and CVD through 11 years of follow-up [8] in the general population. A retrospective study on 616 patients undergoing MHD revealed that lower HGS correlated with increased risks of all-cause mortality and CVE-related hospitalizations [9]. El-Katab et al. indicated that there was a strong correlation between PS and HGS, and that PS might be easier to measure than HGS, perhaps as a more straightforward and convenient screening tool for assessing muscle strength in patients undergoing dialysis [10]. Numerous studies have now demonstrated a strong association between PS and a poor prognosis and unfavorable disease states among chronically ill patients. A cross-sectional study demonstrated that PS is an independent factor significantly associated with mild cognitive impairment in CVD patients [11]. A retr*o*spective cohort study on the relationship between decreased functional status after discharge from and re-admission to the intensive care unit (ICU) reported that patients who were readmitted exhibited poorer functional status and lower PS [12]. A cross-sectional study involving 161 chronic kidney disease (CKD) patients (average age, 70.3 years) showed that muscle weakness measured based on HGS and PS was associated with increased age and decreased appendix muscle mass [13].

Currently, a research gap exists in understanding the relationships among PS, HGS, CVE, and all-cause mortality among individuals undergoing MHD. Therefore, this study aimed to comprehensively examine the impact of PS and HGS on both survival and CVE among patients undergoing MHD in order to offer valuable insights for clinical decision-making.

## Materials & methods

### Study population

This retrospective cohort study was conducted at a single medical center. Overall, 182 participants were collected among individuals who were undergoing MHD at the Hemodialysis Center of Guangzhou Red Cross Hospital in March 2021. Inclusion criteria encompassed an age ≥ 18 years, undergoing hemodialysis thrice weekly for at least 4 h per session, and maintaining regular hemodialysis for over 3 months (*n* = 174). Exclusion criteria included the absence of PS and HGS data, missing follow-up data, patients with pacemakers, concurrent malignancies, recent heart failure, acute myocardial infarction, cerebrovascular accidents, severe infections within the past 3 months, physical disabilities, or inability to cooperate with the study procedures. Ultimately, a total of 140 patients were included in the primary outcome study. Additionally, when studying secondary outcomes (CVE), we excluded patients who already had CVD at baseline, and ultimately 90 patients were included in the study. After 24 months of follow-up, 36 patients developed CVE.

### Clinical data

Demographic data encompassed age, sex, body mass index (BMI), and dialysis vintage. Comorbidity and lifestyle factors considered were hypertension, diabetes, CVD, smoking status, alcohol consumption status, and physical activity level. Before hemodialysis, venous blood samples were collected and promptly delivered to our clinical laboratory within a 2-h timeframe. The laboratory parameters evaluated included hemoglobin, serum calcium, serum phosphorus, serum parathyroid hormone, serum albumin, triglyceride, total cholesterol, low-density lipoprotein cholesterol, high-density lipoprotein cholesterol, interleukin-6, high-sensitivity C-reactive protein, serum creatinine, blood urea nitrogen levels, estimated glomerular filtration rate (eGFR), and urea clearance index (Kt/V). Nutritional risk assessment was conducted using the Nutritional Risk Screening 2002 (NRS-2002) tool [14, 15].

BMI was calculated as weight (kg) divided by the square of height (m^2^). CVE encompassed heart failure, coronary heart disease, unstable angina, myocardial infarction, malignant arrhythmias, and stroke. Activity levels were categorized into low (average non-dialysis daily step count of < 4,000 steps) and moderate-vigorous activities [16]. The eGFR was determined using the isotope dilution mass spectrometry four-variable modification of diet in renal disease study equation [17]: GFR = 175 × standardized Scr ^-1.154 × age^-0.203 × 1.212 (if black) × 0.742 (if female). The urea clearance index of the single-chamber model (spKt/V) was computed using the following formula: spKt/V = -Ln(R-0.008t) + (4-3.5R) × UF/W, in which R represents the post-permeation BUN/pre-permeation BUN ratio, t stands for dialysis time, UF signifies ultrafiltration volume, and W denotes post-permeation weight. An NRS-2002 score ≥ 3 indicated individuals at nutritional risk.

Hemodialysis treatment for the patients was conducted using a Braun Dialog + device (B. Braun Co., Ltd., Melsungen, Germany) in conjunction with a REXEED-15 L high-throughput polysulfone membrane dialyzer (Asahi Kasei Corp., Tokyo, Japan). The dialyzer utilized had a membrane area of 1.5 m^2^, dialysis blood flow rate ranging from 200 to 300 mL/min, dialysis fluid flow rate of 500 mL/min, and dialysis duration of 4 h.

### PS and HGS measurements

Baseline digital pinch and grip force meters (Model 12–0091, Fabrication Enterprises Inc., USA) were used to quantify the patient’s pinch and grip forces. The HGS measurement was conducted in adherence to the recommended standards for seated measurements established by the American Society of Hand Therapists. Each participant’s PS and HGS were gauged thrice on the non-arteriovenous fistula arm, and the resultant average was recorded [18]. During the HGS measurement, the grip distance was appropriately adjusted based on the patient’s hand size. The patient was positioned in a seated stance, with the elbow flexed at 90°, wrist extended within a 0–30° range, and ruler deviation maintained between 0° and 15°. The pinch force meter was positioned between the thumb pad and the radial side of the middle phalanx of the index finger. As this only requires the wrist to be in a neutral position, the measurements can be performed with or without dialysis.

### Follow-up method

The study encompassed an observation period extending from March 2021 to March 2023. Throughout this period, occurrences, such as death, CVE, regression events, and transfer out of our hemodialysis center, were recorded. Additionally, the duration until regression events (in months) was documented. The primary outcome was all-cause mortality, whereas the secondary outcome included recorded CVE during the follow-up period. Patients who transferred out of our hemodialysis center and underwent telephone follow-up and who remained alive upon the conclusion of the observation period marked the endpoints; the total observation duration was 24 months. Data of patients who were lost to follow-up were excluded from the analysis.

### Statistical analysis

Statistical analysis was conducted using SPSS 26.0, R (http://www.R-project.org, Version 4.3.1) with packages “survival,” “survminer,” and “RMS,” as well as Empower Stats software (http://www.empowerstats.com). Continuous variables were presented as mean ± standard deviation or median (P25, P75), whereas categorical variables were expressed as frequency (percentage). Differences in PS and HGS groups were evaluated using χ² tests for categorical variables, analysis of variance tests for normally distributed data, and Kruskal–Wallis H tests for skewed distributions. Kaplan–Meier curves were employed to illustrate mortality and CVE trends across different levels of PS and HGS.Univariate Cox regression and multivariate proportional hazards regression analyses were carried out to identify independent prognostic factors. In the multivariate analysis, we included the variables that were found to be significantly associated with all-cause mortality and CVE in the univariate analysis (Supplementary Table S1). However, we found that there were no confounding variables that met the above criteria, possibly due to the small sample size in this study. Therefore, we explored potential confounding factors from clinical applications and related studies. Subsequently, multivariate Cox proportional hazard regression models were employed to investigate the independent association of PS and HGS with all-cause mortality and CVE. Four models were used: Model 1 was unadjusted; Model 2 was adjusted for age, sex, dialysis age, BMI, hypertension, diabetes, and CVD; Model 3 was adjusted for age, sex, dialysis age, BMI, serum creatinine, and physical activity; and Model 4 was adjusted for age, sex, dialysis age, BMI, low-density lipoprotein cholesterol, serum albumin, and high-sensitivity C-reactive protein. A Cox model with restricted cubic spline functions was utilized to analyze dose-response relationships between PS, HGS, and mortality to enhance robustness and test for trends based on the variable containing a median value for each quartile to validate continuous variable results. A p-value of < 0.05 was considered statistically significant.

## Results

### Baseline characteristics

**Fig. 1:**
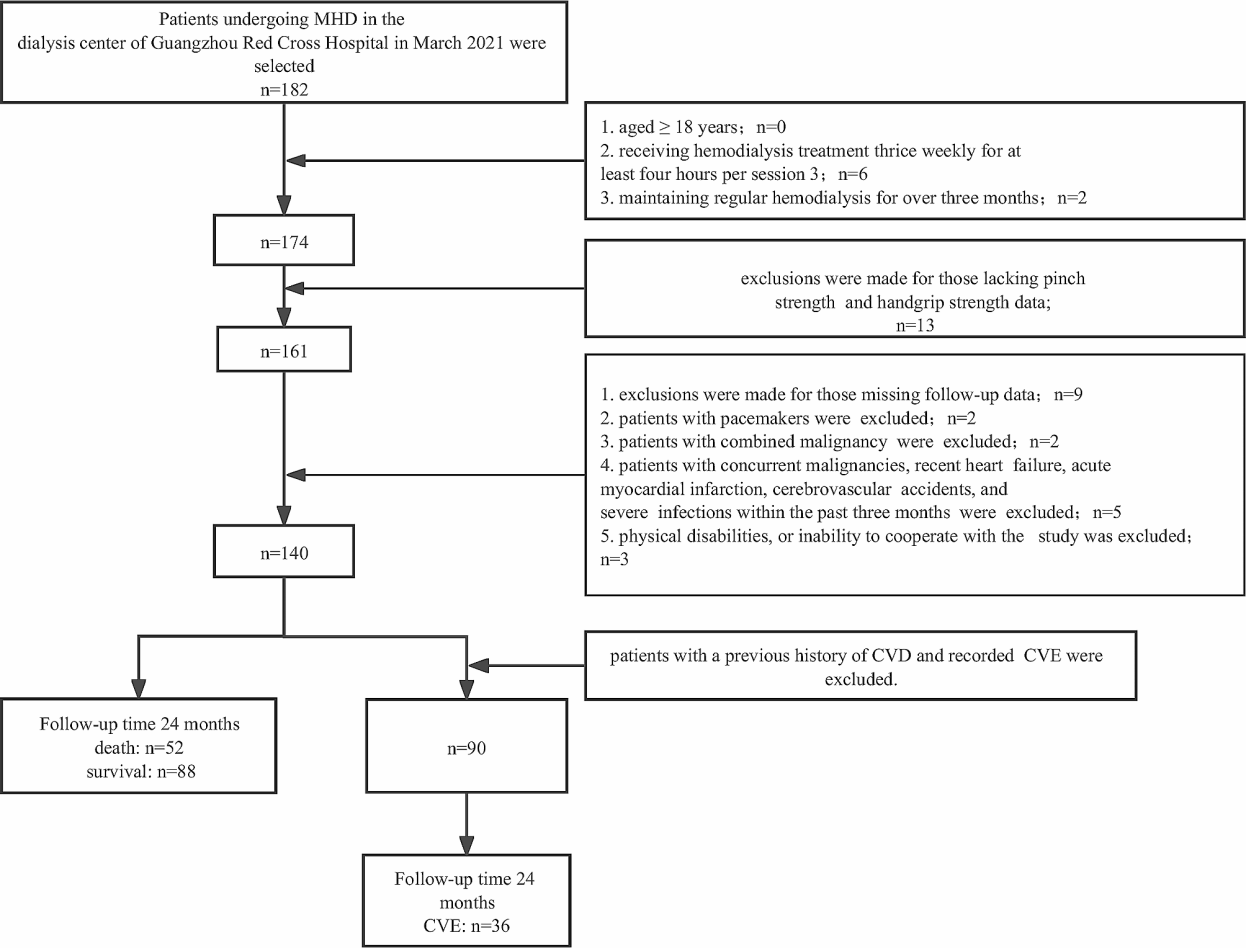
Study population selection flowchart

Overall, 149 patients were included, and 9 patients were lost during follow-up. Figure 1 illustrates the flowchart of the study and outlines the study population selection process. Ultimately, the study enrolled 140 participants (53 men, 87 women) with a mean age of 66.71 ± 12.61 years; 52 deaths (37.14%) occurred during the 24 months of follow-up, collectively culminating in a 2-year survival rate of 63%. Within the same period, a total of 36 CVE transpired, comprising 11 cases of heart failure, 6 of coronary artery disease, 5 of unstable angina, 3 of myocardial infarction, 5 of malignant arrhythmia, and 6 of stroke.

Higher baseline PS and HGS levels were correlated with younger age, female sex, elevated serum creatinine, eGFR, serum phosphorus, serum albumin levels, and a lower risk of malnutrition. Participants in the higher PS and HGS groups also demonstrated lower interleukin-6 levels (all *p* < 0.05), as summarized in Tables 1 and 2.

**Table 1:** Baseline characteristics of the participants by quartiles of PS

Variable	Q1 (2.75–3.60 kg) (*n* = 35)	Q2 (4.40–4.80 kg) (*n* = 29)	Q3 (5.20–6.25 kg) (*n* = 39)	Q4 (7.30–9.10 kg) (*n* = 37)	*p*-value
**Age (years)**	71.91 ± 11.43	70.59 ± 9.61)	67.69 ± 12.80	57.73 ± 11.11	< 0.001
**Sex, n (%)**					< 0.001
Female	14 (40.00)	13 (44.83)	24 (61.54)	36 (97.30)	
Male	21 (60.00)	16 (55.17)	15 (38.46)	1 (2.70)	
**Smoke, n (%)**	6 (17.14)	7 (24.14)	14 (35.90)	19 (51.35)	0.013
**Alcohol consumption status, n (%)**	6 (17.14)	2 (6.90)	12 (30.77)	13 (35.14)	0.027
**Physical activity, n (%)**					0.055
Light	33 (94.29)	29 (100.00)	35 (89.74)	30 (81.08)	
Moderate - Vigorous	2 (5.71)	0 (0.00)	4 (10.26)	7 (18.92)	
**BMI (kg/m** ^**2**^ **)**	23.51 ± 3.98	23.22 ± 3.27	24.20 ± 3.99)	24.04 ± 3.61	0.684
**Hypertension, n (%)**	32 (91.43)	26 (89.66)	36 (92.31)	34 (91.89)	0.983
**Diabetes, n (%)**	19 (54.29)	16 (55.17)	19 (48.72)	15 (40.54)	0.596
**Cardiovascular diseases, n (%)**	19 (54.29)	8 (27.59)	14 (35.90)	9 (24.32)	0.042
**Dialysis age(months)**	31.00 (15.50–51.50)	26.00 (10.00–45.00)	33.00 (19.50–59.50)	24.00 (13.00–57.00)	0.509
**Serum creatinine(µmol/L)**	774.26(645.00-839.50)	769.49(668.00-956.00)	985.21(838.50-1109.50)	1161.92(996.00-1299.00)	< 0.001
**eGFR (mL/min)**	5.42 (4.54–9.30)	7.30 (4.64–10.03)	10.08 (4.46–12.37)	12.69 (11.27–14.69)	< 0.001
**BUN (mmol/L)**	23.97(18.83–27.60)	24.69(20.60–28.40)	31.75(23.55-32.00)	28.95(25.30–33.00)	0.108
**KT/V**	1.47 (1.28–1.69)	1.41 (1.25–1.58)	1.47 (1.15–1.54)	1.25 (1.16–1.36)	0.171
**Bicarbonate (mmol/l)**	20.11(18.38–22.60)	22.58(20.20–23.50)	20.95(19.20-22.95)	20.27(18.90–21.70)	0.058
**Ca (mmol/l)**	2.17 (2.07–2.30)	2.16 (2.00-2.24)	2.21 (2.09–2.37)	2.19 (2.04–2.36)	0.754
**P (mmol/l)**	1.99 (1.71–2.29)	1.90 (1.62–2.29)	2.25 (1.82–2.68)	2.44 (1.96–2.71)	< 0.001
**PTH (pmol/L)**	32.03(14.61–38.36)	25.70(16.50–29.80)	38.25(22.82–47.74)	37.77(25.70-47.81)	0.110
**HDL -C (mmol/L)**	1.21 (0.83–1.42)	1.13 (0.78–1.30)	1.12 (0.77–1.35)	1.12 1 (0.78–1.42)	0.831
**LDL -C (mmol/L)**	2.49 (1.88–2.99)	2.44 (1.83–2.87)	2.44 (1.87–3.05)	2.27 (1.58-3.00)	0.740
**TG (mmol/L)**	2.07 (1.00-2.32)	1.94 (1.00-2.55)	2.03 (1.30–2.50)	1.85 (0.95–2.10)	0.939
**TC (mmol/L)**	4.66 (3.70–5.62)	4.48 (3.62–5.22)	4.42 (3.70-5.00)	4.13 (3.62–4.75)	0.237
**Hemoglobin (g/L)**	96.00(90.00-104.0)	101.00(88.00-112.0)	97.00 (89.00-107.50)	101.00(87.00-113.00)	0.774
**ALB (g/L)**	35.44(32.80–38.00)	34.74(32.00–37.00)	37.12(34.85–39.55)	37.66(35.40–39.60)	< 0.001
**Prealbumin (g/L)**	282.27(240.70-330.10)	493.11(239.80-334.20)	327.63(277.80-366.35)	350.70(324.40-381.20)	0.442
**Nutritional risk**	12 (36.36)	10 (34.48)	9 (24.32)	1 (2.78)	0.004
**IL-6 (pg/mL)**	21.62(7.20-28.73)	13.46 (8.82–17.47)	11.39 (6.01–14.95)	10.26(5.46–10.97)	< 0.001
**Hs-CRP (mg/L)**	10.90(2.38-11.00)	7.78 (1.40–7.33)	6.25 (1.30–7.70)	5.98 (1.50–5.45)	0.235

Note: Values for continuous variables are indicated as mean ± standard deviation or median [P25, P75] and for categorical variables as count (percentage)

Abbreviations: BMI, body mass index; eGFR, estimated glomerular filtration rate; BUN, blood urea nitrogen; KT/V, urea clearance index; Ca, serum calcium; P, serum phosphorus; PTH, parathyroid hormone; HDL-C, high-density lipoprotein cholesterol; LDL-C, low-density lipoprotein cholesterol; TG, triglycerides; TC, total cholesterol; ALB, serum albumin; IL-6, interleukin-6; Hs-CRP, high-sensitivity C-reactive protein

**Table 2:** Baseline characteristics of the participants by quartiles of HGS

Variables	Q1 (5.90–9.80 kg) (*n* = 35)	Q2 (13.05–15.80 kg) (*n* = 35)	Q3 (18.25–21.50 kg) (*n* = 35)	Q4 (25.50–31.90 kg) (*n* = 35)	*p*-value
**Age (years)**	73.91 ± 10.92	70.06 ± 10.72	65.74 ± 10.92	57.14 ± 11.68)	< 0.001
**Sex, n (%)**					< 0.001
Female	11 (31.43)	15 (42.86)	28 (80.00)	33 (94.29)	
Male	24 (68.57)	20 (57.14)	7 (20.00)	2 (5.71)	
**Smoking status, n (%)**	9 (25.71)	7 (20.00)	14 (40.00)	16 (45.71)	0.861
**Alcohol consumption status, n (%)**	7 (20.00)	8 (22.86)	8 (22.86)	10 (28.57)	0.027
**Physical activity, n (%)**					0.015
Light or none	34 (97.14)	35 (100.00)	29 (82.86)	29 (82.86)	
Moderate -vigorous	1 (2.86)	0 (0.00)	6 (17.14)	6 (17.14)	
**BMI (kg/m** ^**2**^ **)**	23.96 ± 3.20	23.40 ± 4.85	23.26 ± 3.16	24.50 ± 3.48	0.492
**Hypertension, n (%)**	32 (91.43)	34 (97.14)	31 (88.57)	31 (88.57)	0.534
**Diabetes, n (%)**	19 (54.29%)	19 (54.29%)	16 (45.71%)	15 (42.86%)	0.692
**Cardiovascular diseases, n (%)**	20 (57.14%)	14 (40.00%)	9 (25.71%)	7 (20.00%)	0.006
**Dialysis age(months)**	29.00 (14.00–48.00)	27.00 (12.00–48.00)	29.00 (11.50–55.00)	39.00 (16.50–61.00)	0.817
**Serum creatinine(µmol/L)**	738.50 (615.50–867.50)	801.00 (707.50–992.00)	963.00 (852.50–1095.00)	1165.00 (1026.50–1329.50)	< 0.001
**eGFR (mL/min)**	5.41 (4.46–7.91)	7.14 (4.46–10.00)	10.26 (8.09–12.23)	12.84 (11.56–15.04)	< 0.001
**BUN (mmol/L)**	22.30 (18.43–25.88)	27.30 (22.40–32.00)	26.20 (23.55–30.60)	29.10 (26.00–33.35)	0.059
**KT/V**	1.42 (1.28–1.71)	1.37 (1.21–1.65)	1.32 (1.20–1.45)	1.29 (1.16–1.36)	0.033
**Bicarbonate (mmol/L)**	21.00 (19.55–23.20)	20.20 (18.20–21.95)	20.90 (19.45–24.15)	20.30 (19.05–22.80)	0.155
**Ca (mmol/L)**	2.13 (2.05–2.27)	2.21 (2.04–2.38)	2.15 (2.02–2.25)	2.17 (2.05–2.38)	0.516
**P (mmol/L)**	1.96 (1.66–2.18)	2.10 (1.73–2.33)	2.34 (1.98–2.56)	2.44 (1.83–2.95)	< 0.001
**PTH (pmol/L)**	24.95 (14.80–34.69)	26.79 (16.41–34.43)	31.93 (21.78–49.62)	33.64 (24.48–48.11)	0.126
**HDL-C (mmol/L)**	1.08 (0.80–1.31)	1.14 (0.78–1.46)	1.12 (1.01–1.47)	0.92 (0.77–1.27)	0.268
**LDL-C (mmol/L)**	2.36 (1.82–3.14)	2.19 (1.77–2.92)	2.29 (1.91–2.73)	2.31 (1.62–2.80)	0.414
**TG (mmol/L)**	1.70 (1.20–2.20)	1.80 (1.00–2.30)	1.30 (1.00–2.42)	1.50 (1.10–2.50)	0.777
**TC (mmol/L)**	4.70 (3.70–5.45)	4.40 (3.60–5.55)	4.30 (3.82–4.80)	4.30 (3.70–4.60)	0.206
**Hemoglobin (g/L)**	95.00 (84.00–103.00)	102.00 (91.50–112.50)	101.00 (89.50–113.00)	98.00 (85.95–109.50)	0.183
**ALB (g/L)**	34.95 (32.35–37.92)	35.90 (34.05–37.35)	36.80 (34.20–38.75)	39.10 (36.75–40.00)	< 0.001
**Prealbumin (g/L)**	278.60 (230.95–317.25)	305.40 (245.75–339.50)	323.40 (263.80–359.05)	358.90 (327.35–396.10)	0.410
**Nutritional risk**	15 (42.86%)	9 (28.12%)	6 (17.65%)	2 (5.88%)	0.003
**IL-6 (pg/mL)**	11.02 (6.87–19.36)	20.02 (10.76–27.99)	8.74 (5.70–11.83)	6.58 (4.70–10.07)	< 0.001
**Hs-CRP (mg/L)**	6.70 (2.75–9.35)	5.20 (1.95–12.85)	2.80 (1.30–7.60)	2.20 (1.30–5.00)	0.293

Note: Values for continuous variables are indicated as mean ± standard deviation or median [P25, P75] and for categorical variables, as count (percentage)

Abbreviations: BMI, body mass index; eGFR, estimated glomerular filtration rate; BUN, blood urea nitrogen; KT/V, urea clearance index; Ca, serum calcium; P, serum phosphorus; PTH, parathyroid hormone; HDL-C, high-density lipoprotein cholesterol; LDL-C, low-density lipoprotein cholesterol; TG, triglycerides; TC, total cholesterol; ALB, serum albumin; IL-6, interleukin-6; Hs-CRP, high-sensitivity C-reactive protein

### Kaplan–Meier curves

**Fig. 2:**
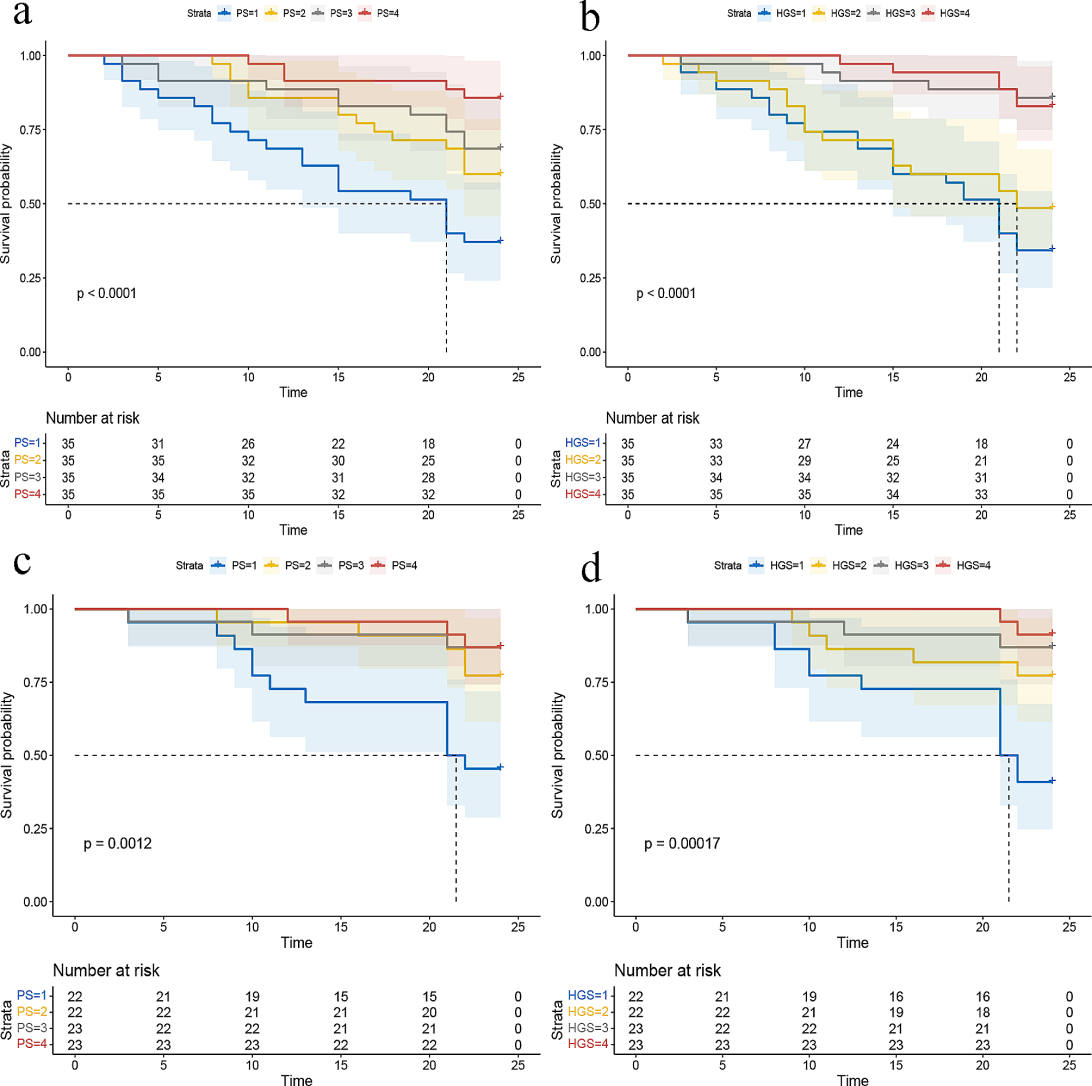
Survival curves for patients with different levels of PS or HGS and all-cause mortality or CVE. Figure 2. Survival curves for patients with different levels of PS or HGS; (**a**) all-cause mortality by PS quartiles (Q); (**b**) all-cause mortality by HGS quartiles (Q); (**c**) CVE by PS quartiles (Q); (**d**) CVE by HGS quartiles (Q). Abbreviation: PS, pinch strength; HGS, handgrip strength; CVE, cardiovascular events

We observed a gradual decrease in the unadjusted risk for both all-cause mortality and CVE as the quartiles of PS and HGS increased, as depicted in Fig. 2.

### Association with outcomes

#### All-cause mortality

The median PS levels among participants who died and remained alive during the follow-up were 4.40 (3.48–5.35) kg and 6.00 (4.70–7.62) kg, respectively. Each 1-standard deviation (SD) increase in PS value was associated with a 29% lower hazard of death (HR, 0.71; 95%CI, 0.61–0.84) in the unadjusted model. In Model 2, each 1-SD increase in PS value was associated with a 21% lower hazard of death (HR, 0.79; 95% CI, 0.65–0.96). In Model 3, each 1-SD increase in PS value was associated with a 20% lower hazard of death (HR, 0.80; 95% CI, 0.65–0.97). In Model 4, the association persisted, with a 22% lower hazard of death for each 1-SD increase in PS value (HR, 0.78; 95% CI, 0.64–0.95; Table 3).

The median HGS levels among participants who died and remained alive during the follow-up were 13.32 (9.15–16.77) kg and 19.85 (15.15–25.43) kg respectively. Each 1-SD increase in HGS value was associated with an 8% lower hazard of death (HR, 0.92; 95% CI, 0.88–0.95) in the unadjusted model. In Model 2, 3, each 1-SD increase in HGS value was associated with a 21% lower hazard of death (HR, 0.94; 95% CI, 0.90–0.99). In Model 4, each 1-SD increase in HGS value was associated with a 8% lower hazard of death (HR, 0.92; 95% CI, 0.88–0.97).

**Table 3:** Multivariate Cox regression models for evaluating the impacts of PS and HGS on all-cause mortality and cardiovascular events

	PS		HGS	
	HR(95% CI)	*p*-value	HR(95% CI)	*p*-value
**All-cause mortality**				
Model 1	0.71 (0.61–0.84)	< 0.001	0.92 (0.88–0.95)	< 0.001
Model 2	0.79 (0.65–0.96)	0.018	0.94 (0.90–0.99)	0.013
Model 3	0.80 (0.65–0.97)	0.024	0.94 (0.90–0.99)	0.012
Model 4	0.78 (0.64–0.95)	0.013	0.92 (0.88, 0.97)	0.002
**Cardiovascular events**				
Model 1	0.90 (0.77–1.05)	0.183	0.95 (0.91–0.99)	0.009
Model 2	0.98 (0.80–1.21)	0.847	0.95 (0.90–1.00)	0.049
Model 3	1.01 (0.81–1.26)	0.940	0.95 (0.90–1.01)	0.076
Model 4	0.95 (0.76–1.19)	0.680	0.95 (0.89–1.01)	0.089

Note: HR, hazard ratio; 95% CI, 95% confidence interval; PS, pinch strength; HGS, handgrip strength; BMI, body mass index

Model 1 was unadjusted;

Model 2 was adjusted for age, sex, dialysis age, BMI, hypertension, diabetes, and cardiovascular disease;

Model 3 was adjusted for age, sex, dialysis age, BMI, serum creatinine, and physical activity;

Model 4 was adjusted for age, sex, dialysis age, BMI, low-density lipoprotein cholestero, serum albumin, and high-sensitivity C-reactive protein

#### CVE

For participants who experienced and did not experience a CVE, the median PS levels were 5.15 (4.40–6.72) kg and 6.00 (4.45–7.80) kg, respectively. However, our study revealed that PS was not significantly associated with CVE (Table 3).

The median HGS level was 15.20 (12.15–21.05) kg among participants who experienced a CVE and 20.50 (15.35–27.25) kg among those who did not. In the unadjusted models, there was a 5% decrease in the hazard of CVE for each 1-SD increase in HGS value (HR, 0.95; 95% CI, 0.91–0.99). However, in Models 2, 3, and 4, this association did not exist.

### Categorical models and splines

We found evidence supporting a linear correlation between increased quartiles of PS levels and all-cause mortality in the main multivariate Models 2 (p for trend = 0.040), 3 (p for trend = 0.045), and 4 (p for trend = 0.021). Similar patterns were observed for HGS in the main multivariate Models 2 (p for trend = 0.023), 3 (p for trend = 0.019), and 4 (p for trend = 0.020) (Table 4).

**Table 4:** Adjusted hazard of all-cause mortality by PS and HGS level quartiles

	Death (*N*)	Alive (*N*)	Model 1	Model 2	Model 3	Model 4
			HR (95% CI)	HR (95% CI)	HR (95% CI)	HR (95% CI)
**PS (kg, median [P25, P75])**						
Q1 (3.30 [2.75–3.60])	22	13	1.00 (Ref.)	1.00 (Ref.)	1.00 (Ref.)	1.00 (Ref.)
Q2 (4.60 [4.40–4.80])	13	22	0.47 (0.23–0.98)	0.65 (0.30–1.42)	0.49 (0.23–1.04)	0.58 (0.26–1.29)
Q3 (6.00 [5.20–6.25])	12	23	0.42 (0.21–0.82)	0.56 (0.27–1.16)	0.56 (0.27–1.17)	0.55 (0.25–1.21)
Q4 (8.50 [7.30–9.10])	5	30	0.14 (0.05–0.38)	0.30 (0.10–0.91)	0.31 (0.10–1.01)	0.31 (0.10–1.02)
**P for trend**			< 0.001	0.040	0.045	0.021
**HGS (kg, median [P25, P75])**						
Q1 (6.55 [5.90–9.80])	23	12	1.00 (Ref.)	1.00 (Ref.)	1.00 (Ref.)	1.00 (Ref.)
Q2 (17.40 [13.05–15.80])	18	17	0.72 (0.39– 1.34)	0.78 (0.40–0.51)	0.77 (0.40–1.48)	0.68 (0.34–1.37)
Q3 (20.10 [18.25–21.50])	5	30	0.15 (0.06–0.41)	0.18 (0.06–0.55)	0.19 (0.06–0.54)	0.21 (0.07–0.64)
Q4 (27.60 [25.50–31.90])	6	29	0.18 (0.07–0.44)	0.30 (0.10–0.90)	0.32 (0.10–0.99)	0.30 (0.10–0.95)
**P for trend**			< 0.001	0.023	0.019	0.020

Note: HR, hazard ratio. 95% CI, 95% confidence interval. Ref, reference; PS, pinch strength, HGS, handgrip strength; BMI, body mass index

Model 1 was unadjusted;

Model 2 was adjusted for age, sex, dialysis age, BMI, hypertension, diabetes, and cardiovascular disease;

Model 3 was adjusted for age, sex, dialysis age, BMI, serum creatinine, and physical activity;

Model 4 was adjusted for age, sex, dialysis age, BMI, low-density lipoprotein cholesterol, serum albumin, and high-sensitivity C-reactive protein

### Restricted cubic spline curves

**Fig. 3:**
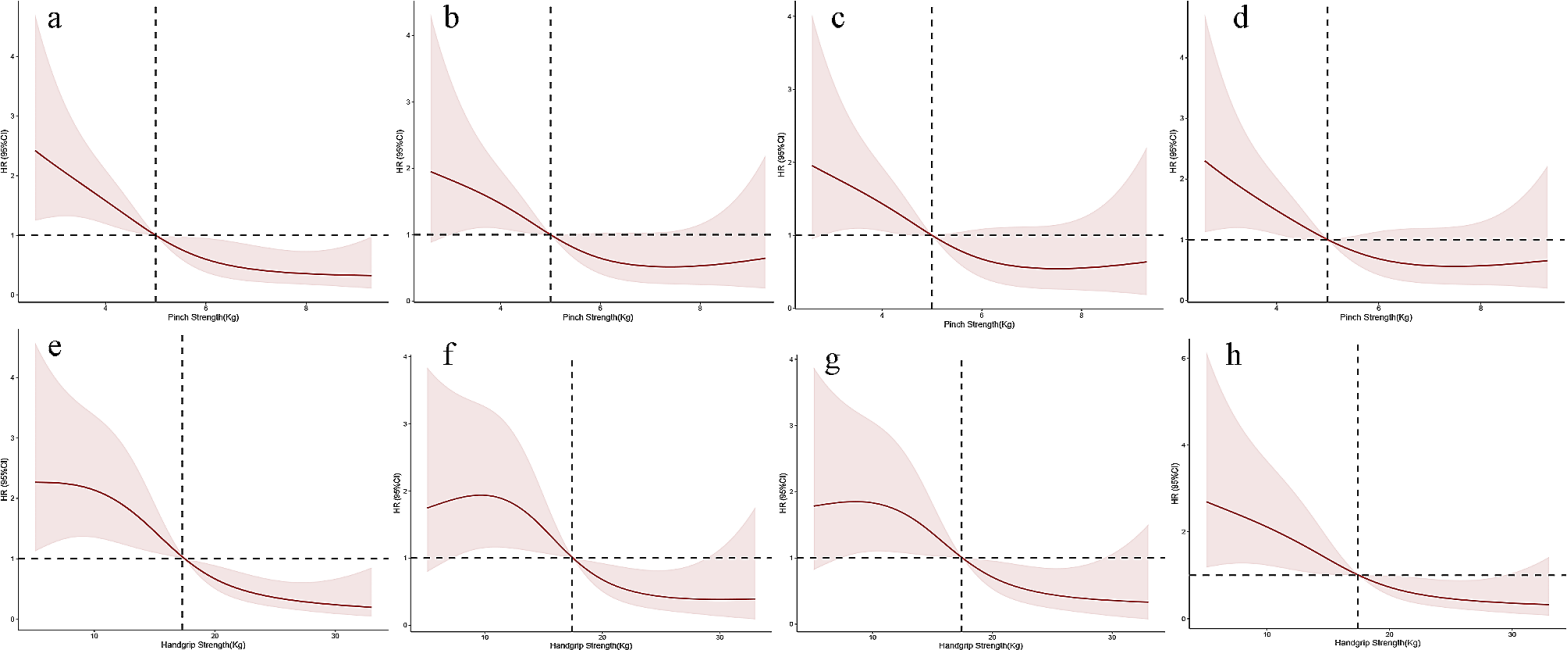
Restricted cubic spline curves for the relationship between PS or HGS and all-cause mortality. Figure 3. Restricted cubic spline curves for the relationship between PS and all-cause mortality in (**a**) Model 1, (**b**) Model 2, (**c**) Model 3, and (**d**) Model 4; similarly, the relationship between HGS and all-cause mortality in (**e**) Model 1, (**f**) Model 2, (**g**) Model 3, and (**h**) Model 4. Note: PS, pinch strength; HGS, hand grip strength, BMI, body mass index Model 1 was unadjusted; Model 2 was adjusted for age, sex, dialysis age, BMI, hypertension, diabetes, cardiovascular disease; Model 3 was adjusted for age, sex, dialysis age, BMI, serum creatinine, and physical activity; Model 4 was adjusted for age, sex, dialysis age, BMI, low-density lipoprotein cholesterol, serum albumin, and high-sensitivity C-reactive protein

Monotonic relationships between PS and HGS levels and all-cause mortality were confirmed by spline analysis results (Fig. 3).

## Discussion

### PS, HGS, and all-cause mortality in patients undergoing MHD

This study aimed to examine the impact of PS and HGS on both survival and CVE among patients undergoing MHD. Our study confirmed that following adjustment for potential confounding factors, both Cox regression analyses and dose-response relationships validated the notion that elevated PS and HGS are associated with a reduction in mortality rate among patients undergoing MHD. This finding aligns with the outcomes of a prior investigation by Cácia Mendes Matos et al., which explored the association between baseline HGS and all-cause mortality in male and female patients undergoing hemodialysis [19]. Notably, our study contributes a novel insight by revealing that PS, in particular, independently predicts all-cause mortality in patients undergoing MHD. When further assessing the dose-response relationship between PS and all-cause mortality, we observed a significant reduction in all-cause mortality in MHD patients when PS exceeded 4.99 kg. The inflection point of PS can be considered a therapeutic target in our clinical work for outcome assessment. Considering that PS measurement is more practical and convenient than HGS measurement in MHD patients, our research findings are of great significance for the development of clinical work [10]. However, as our study is a cross-sectional study, further intervention studies on PS testing are needed to confirm the above relationship, which opens the door to incorporating PS into clinical practice.

Sarcopenia is a progressive and generalized loss of muscle mass and strength/function that occurs with aging [20]. It is strongly associated with poor outcomes in patients with CKD, particularly MHD, and is associated with a significant increase in hospitalization and mortality rates [21]. Sarcopenia diagnosis is usually assessed from a combination of muscle mass, muscle strength, and physical performance [22], and muscle mass assessment needs to be performed using dual-energy X-ray absorptiometry or bioelectrical impedance analysis, which increases cumbersomeness and the cost of operation in the routine management of chronic diseases. PS and HGS can be used as an index for assessing muscle strength [5, 10], providing patients undergoing dialysis with an easier and less budgetary screening tool for muscle strength. HGS was utilized to reflect the energetic and functional status of proteins in CKD patients and can also serve as an independent predictor of numerous unfavorable prognoses [7, 8, 23–25]. Currently, HGS has been shown to predict poor prognosis in the population undergoing MHD. Particularly, Silva et al. and Matos et al. demonstrated that lower HGS values were independently associated with higher malnutrition and a higher risk of death in patients undergoing hemodialysis [19, 26]. Ishihara et al. conducted a cross-sectional study of 135 patients with CVD and proposed that PS was significantly and independently associated with mild cognitive impairment [11]. PS and HGS can also be used to assess activities of daily living in patients with stroke [27], and a retr*o*spective cohort study of patients in the ICU revealed that readmitted patients showed poorer functional status and lower PS [12], all of which show that PS can also serve as a predictor of poor prognosis. However, the number of studies on PS, which is also used as an indicator for assessing muscle strength in the populations undergoing MHD [5, 10, 28], is very limited in comparison to current studies on HGS, which calls for a significant amount of future exploration on this topic. Therefore, the present study expands previous studies by reporting a new finding that confirms that PS and HGS are highly associated with poor prognosis in patients undergoing MHD.

### PS, HGS, and CVE in patients undergoing MHD

Our study did not show a significant association between PS, HGS, and CVE in patients undergoing MHD. Past research consistently indicated that sarcopenia increased vulnerability to CVD, with muscle health being an independent predictor of adverse CVE. Ke Gao et al. used CHARLS data to examine sarcopenia’s correlation with CVD in middle-aged and older adults, finding a higher likelihood of new-onset CVD in those with probable sarcopenia (HR, 1.22, 95% CI, 1.05–1.43) and sarcopenia (HR, 1.33, 95% CI, 1.04–1.71) compared with those without sarcopenia [29]. Moreover, Lopez-Jaramillo et al. suggested that enhancing muscle strength mitigates cardiovascular risk factors [30]. For CKD, a meta-analysis by Wathanavasin et al. supported sarcopenia’s association with increased death risk (adjusted odd ratio [OR], 1.83; 95% CI, 1.40–2.39) and CVE (adjusted OR, 3.80; 95% CI, 1.79–8.09) [31]. Possible explanations for our findings encompass the limitations of using PS and HGS as sole indicators of muscle strength, the potential for data collection errors, the relatively brief follow-up period, and the restricted sample size within our center. In order to comprehensively investigate the relationships between PS, HGS, and CVE, it is imperative that future cohort studies with larger sample sizes and more extended follow-up periods be undertaken.

### Mechanisms of associations between muscle strength and prognosis in patients undergoing MHD

Mechanisms underlying the correlation between PS, HGS, and adverse prognosis in patients undergoing MHD remain unclear. Several factors could contribute to the association among them. First, a study by Ronit et al. demonstrated an independent association between low serum album levels and CVD, aligning with our findings in which higher serum album levels were observed in individuals with elevated PS and HGS. Second, the impact of inflammatory cytokines such as IL-6 and CRP is worth considering; these cytokines exacerbate atherosclerosis and insulin resistance [32], which are key mechanisms in CVD development. Insulin resistance correlates with CVD and contributes to kidney function decline in CKD patients [33], and CVD accelerates mortality in CKD patients [3]. A study in the US associated lower systemic immune-inflammation with increasing HGS [34], consistent with our observation of lower IL-6 levels in those with higher PS and HGS. Third, inflammatory-oxidative stress can lead to muscle wasting by impairing anabolism and increasing proteolysis [35]. Inflammatory factors promote muscle protein degradation through the ubiquitin-proteasome system, caspase-3 apoptotic system, and autophagy-lysosomal pathway [36]. Extensive literature underscores the strong relationship among sarcopenia, mortality, and CVE in patients undergoing MHD [31]. Thus, PS and HGS, as indicators of muscle strength, can offer enhanced predictive value for poor outcomes.

### Limitations

Nevertheless, this study has some limitations. First, the generalization of findings requires caution owing to the single-center data source. Second, PS and HGS were only measured at baseline, constraining our understanding of how temporal changes affect their correlation with mortality and cardiovascular risk. Third, exercise habits and types were not documented, although these could potentially influence PS, HGS, mortality, and cardiovascular outcomes. Finally, despite rigorous record review, the potential for data inaccuracies and unaccounted confounders remains.

## Conclusion

Muscle health displayed associations with both mortality and cardiovascular disease (CVD) in individuals undergoing maintenance hemodialysis (MHD). Notably, both PS and HGS emerged as independent risk factors for all-cause mortality. Unfortunately, our study did not establish an independent correlation between PS, HGS, and CVE. Given the heightened mortality rate among patients undergoing MHD, our findings carry substantial clinical implications, emphasizing the predictive utility of PS and HGS in enhancing patient prognosis. Furthermore, PS’s acceptance by patients due to its compact, straightforward, and easily measurable nature suggests its prospective application in clinical settings as a therapeutic target for condition assessment. To solidify the potential benefits of bolstering PS in prognostic outcomes for patients undergoing MHD, it is imperative to conduct further comprehensive research. This should encompass multicenter studies with larger sample sizes and intervention-based investigations utilizing the PS test.

### Electronic supplementary material

Below is the link to the electronic supplementary material.

Supplementary Material 1

## Data Availability

Charts, plots and tables summarise larger amounts of data. The datasets generated and/or analysed during the current study are not publicly available due [REASON WHY DATA ARE NOT PUBLIC] but are available from the corresponding author on reasonable request.
