# Supplementary material for: Associations between pinch strength, cardiovascular events and all-cause mortality in patients undergoing maintenance hemodialysis
Source: BMC Nephrol. 2024 May 2;25:150. doi: 10.1186/s12882-024-03587-x (PMC11064367; doi:10.1186/s12882-024-03587-x)
Supplement: Supplementary file 1 — Supplementary Material 1 [file 12882_2024_3587_MOESM1_ESM.docx]

**Supplementary Table. Univariate analysis for** **all-cause mortality and cardiovascular events.**

| **Characteristics** | **All-cause mortality** | **Cardiovascular events** |
| --- | --- | --- |
|  | **HR（95%CI）** | **HR（95%CI）** |
| **Age (years)** | 1.05（1.03-1.08） | 1.03（1.00-1.06） |
| **Sex（Female vs Male）** | 0.54（0.31-0.93） | 0.63（0.33-1.23） |
| **Smoking status（YES vs NO）** | 0.68（0.37-1.26） | 1.38（0.68-2.81） |
| **Alcohol consumption status（YES vs NO）** | 0.79（0.48-1.75） | 2.93（1.46-5.87） |
| **Physical activity, （Moderate -Vigorous vs Light or none v）** | 0.32（0.08-1.34） | 0.50（0.12-2.o6） |
| **BMI (kg/m2)** | 0.99（0.92-1.07） | 1.00（0.92-1.10） |
| **Hypertension（YES vs NO）** | 1.68（0.52-5.37） | 5.97（0.82-43.58） |
| **Diabetes （YES vs NO）** | 1.58（0.91-2.75） | 1.14（0.59-2.23） |
| **Cardiovascular diseases （YES vs NO）** | 2.99（1.73-5.18） | - |
| **Dialysis age（months）** | 1.00（0.99-1.01） | 1.00（0.99-1.01） |
| **Creatinine（μmol/L)** | 1.00（0.98-1.00） | 1.00（0.99-1.00） |
| **eGFR (mL/min)** | 0.92（0.86-0.99） | 0.94（0.87-1.00） |
| **BUN (mmol/L)** | 0.95（0.91-0.97） | 1.00（0.95-1.06） |
| **KT/V** | 1.05（0.0.66-1.68） | 1.00（0.99-1.00） |
| **Bicarbonate (mmol/L)** | 0.98（0.91-1.05） | 0.98（0.92-1.06） |
| **Ca (mmol/L)** | 0.90（0.23-3.26） | 1.2（0.28-5.62） |
| **P (mmol/L)** | 0.64（0.41-1.01） | 0.81（0.47-1.40） |
| **PTH (pmol/L)** | 0.90（0.97-1.01） | 1.00（0.99-1.01） |
| **HDL-C (mmol/L)** | 0.96（0.51-1.79） | 1.22（0.63-1.29） |
| **LDL-C (mmol/L)** | 1.22（0.87-1.72） | 0.83（0.53-1.29） |
| **TG (mmol/L)** | 1.00（0.82-1.22） | 0.94（0.72-1.24） |
| **TC (mmol/L)** | 1.20（0.91-1.57） | 0.92（0.66-1.30） |
| **Hemoglobin (g/L)** | 0.98（0.98-1.03） | 0.97（0.98-1.01） |
| **ALB (g/L)** | 0.91（0.84-0.98） | 0.93（0.85-1.00） |
| **Prealbumin (g/L)** | 1.00（1.00-1.01） | 0.96（0.99-1.00） |
| **Nutritional risk （≥3 vs <3）** | 2.06（1.13-3.74） | 1.21（0.55-2.55） |
| **IL-6 (pg/mL)** | 1.01（0.99-1.03） | 1.00（0.97-2.06） |
| **Hs-CRP (mg/L)** | 1.01（0.99-1.04） | 0.97（0.93-1.01） |

Abbreviations: BMI, body mass index; eGFR, glomerular filtration rate; BUN, blood urea nitrogen; KT/V, urea clearance index; Ca, serum calcium; P, serum phosphorus; PTH, parathyroid hormone; HDL-C, high-density lipoprotein cholesterol; LDL-C, low-density lipoprotein cholesterol; TG, triglycerides; TC, total cholesterol; ALB, serum albumin; IL-6, interleukin-6; Hs-CRP, high-sensitivity C-reactive protein.
